# The Potential of Fasting and Caloric Restriction to Mitigate Radiation Damage—A Systematic Review

**DOI:** 10.3389/fnut.2020.584543

**Published:** 2020-09-18

**Authors:** Simon Valayer, David Kim, Anna Fogtman, Ulrich Straube, Andrew Winnard, Nick Caplan, David A. Green, Flora H. P. van Leeuwen, Tobias Weber

**Affiliations:** ^1^European Space Agency (ESA), European Astronaut Center (EAC), Space Medicine Team (HRE-OM), Cologne, Germany; ^2^Faculty of Medicine Paris VI, Sorbonne University, Paris, France; ^3^Faculty of Medicine, University of British Columbia, Vancouver, BC, Canada; ^4^Faculty of Health and Life Sciences, Northumbria University, Newcastle upon Tyne, United Kingdom; ^5^KBR GmbH, Cologne, Germany; ^6^Center of Human & Applied Physiological Sciences (CHAPS), King's College London, London, United Kingdom; ^7^Faculty of Medicine, Utrecht University, Utrecht, Netherlands

**Keywords:** fasting, caloric restriction, radio-protection, SIRTUIN, irradation, space flight, deep space, radiology

## Abstract

Detrimental health effects from ionizing radiation to living organisms is one of the key concerns identified and addressed by Radiation Protection institutions, nationally and internationally on Earth and for human spaceflight. Thus, new methods for mitigating the adverse effects of ionizing radiation are urgently needed for terrestrial health and deep space exploration. Caloric restriction and (intermittent-) fasting have been reported to elicit a variety of immediate and long-term physiological effects. The rapidly growing body of evidence of research studies investigating the effects of caloric restriction and dietary fasting points toward a multitude of benefits affecting numerous physiological systems. Therefore, a systematic review was performed to evaluate the evidence of caloric restriction and dietary fasting on the physiological response to ionizing radiation in humans and animals. All experimental studies of humans, animals, and eukaryotic cell lines available in PubMed, Cochrane library, and specialized databases were searched comparing irradiation post-caloric restriction or fasting to a non-nutritionally restricted control group on a broad range of outcomes from molecular to clinical responses. The initial search yielded 2,653 records. The final analysis included 11 studies. Most studies investigated survival rate or cancer occurrence in animals. Included studies did not reveal any benefit from pre exposure caloric restriction, except when performed with post radiation caloric restriction. However, the effects of pre-exposure fasting suggest increased resilience to ionizing radiation.

## Introduction

Ionizing radiation has numerous negative biological effects on almost all living organisms (1). Humans may be challenged not only by classical background radiation and nuclear events/disasters but also *via* exposure to radiotherapy, air travel or even space travel. Over the past few decades, the field of radiation protection has made significant progress in devising methodologies to protect humans from ionizing radiation including development of regulations relating to the exposure to radiation use in medical, civilian, and military contexts (2).

However, greater knowledge of the mechanisms that determine the biological damage resulting from ionizing radiation is required in order to inform the development of more effective radiotherapy delivery methodologies and protective countermeasures. Terrestrially, this is of critical importance in radiotherapy in order to reduce secondary tissue damage, while maximizing radiation delivery to cancerous tissues. Furthermore, mitigating the negative effects of ionizing radiation is arguably the most significant challenge that must be addressed to facilitate human space exploration, beyond the relative protection provided by the Earth's magnetic field in low earth orbit (3).

Ionizing radiation is known to induce significant damage to cells. For instance, ionizing radiation exposure is reported to induce pathological states including acute radiation syndrome (4), (solid and non-solid) cancer (5), and organ dysfunction (e.g., radiation pneumonitis, radiation enteritis) (6, 7). The occurrence of some pathological states are directly dependent on the total received radiation dose (deterministic effects) (8), while other pathological states such as cancer appear to have probabilistic increases with dose (stochastic effects) (9).

The physiological effects of ionizing radiation exposure can be categorized as either direct or indirect (10). Direct effects refer to the immediate damage ionizing radiation induces on DNA within cellular nuclei such as single or double strand breakages in DNA. Indirect effects characterize the interaction between ionizing radiation and other molecules, such as water. In contrast, the so-called “bystander effect” is defined as the biological response observed in cells without direct exposure to radiation. For instance, indirect effects generate free radicals and reactive oxygen species that create an environment of increased genetic instability leading to indirect damage to DNA and other cellular components (10). In normal physiologic states, cellular activity generating oxidative stress *via* production of free radicals and ROS is mitigated by the antioxidant system (11) *via* the donation of electrons by antioxidants. Thus, the balance between free radical production and antioxidant activity determines the cellular oxidative stress. Thus, stimulation of increased antioxidant activity is likely to reduce oxidative stress, and resultant DNA damage, thereby potentially mitigating at least some of the damage induced by ionizing radiation.

Interestingly, research has shown that fasting (defined as the complete absence of food for >12 h) can extend rodent lifespans (12, 13). Furthermore, caloric restriction—defined as reduced caloric intake for more than 12 h—can also extend rodent (14, 15) and primates lifespans (16). Whilst several physiological mechanisms have been proposed over the years, more recently, there is increasing evidence that fasting and caloric restriction can directly modulate and reduce cellular oxidative stress, which may underpin lifespan extension (17, 18). Such mechanisms may also potentially reduce oxidative stress and thus represent a biological countermeasure to ionizing radiation. Considering the above, in addition to reducing ionizing radiation one possibility to reduce the overall risk of developing dose-dependent or stochastic radiation-induced medical conditions could be to reduce the radiation-sensitivity of cells, thus increasing their radiation resilience.

Human research investigating the effects of fasting and caloric restriction on radio-protective mechanisms seems to be scarce. An *in vitro* pilot study utilizing human serum showed an increased oxidative stress resistance after caloric restriction (19). Other studies have tried to describe cellular mechanisms responsible for the link between food intake and oxidative stress and have provided potential explanations through the interaction with energy sensing pathways (20, 21). Proteins such as Sirtuins (22–27), FOXO (28), TOR (29), AMPK (30), or NRF2 (31) seem to be key players in the mediation of stress resistance and anti-oxidant response to fasting and caloric restriction. Based on these findings, it could be suggested that caloric restriction or fasting might mitigate biological damage induced by secondary effects of ionizing radiation, through positive interaction with the cellular antioxidant system while also decreasing incidence of diseases like cancer (32). Thus, the aim of this review was to evaluate the evidence for fasting and/or caloric restriction as an approach to radioprotection. For terrestrial applications, there are various implications of exploring the protection that could be provided to patients undergoing radiation therapy or medical imaging, to those exposed to higher than baseline radiation from occupational sources, and to accidental radiation exposure incidents.

## Materials and Methods

### Terminology

The terms caloric restriction and fasting are sometimes conflated. Fasting is typically defined in the literature as no, or minimal, caloric intake for at least 12 h (33). Moreover, fasting and caloric restriction can be applied in a number of ways (e.g., intermittent fasting, periodic fasting, continuous fasting, continuous caloric restriction, or intermittent caloric restriction). This review defines “fasting” as a complete absence of food for longer than 12 h, while the term “caloric restriction” refers to reduced caloric intake (compared to normal) for longer than 12 h. Such term do not include “starvation” which relates to a chronic nutritional insufficiency and thus is beyond the scope of the present study.

### Search Strategy

The guidelines in the Cochrane handbook (www.cochrane.com; version 5.1) were followed using tools created by the Aerospace Medicine Systematic Review Group (AMSRG: http://aerospacemed.rehab/systematic-review-group) for data extraction, quality assessment of studies, and effect size calculations. Furthermore, this review followed the guidelines of the Preferred Reporting Items for Systematic Review and Meta-Analyses (PRISMA). PubMed, Embase, Cochrane Library and space agencies' local databases (NASA, ESA, and DLR) were searched for eligible studies published before November 30th, 2018. The detailed search strategy using Boolean logic is shown in Supplementary Table 1. Due to the lack of an advanced research tool using Boolean logic in NASA's, ESA's, and DLR's internal archives, the search strategy was adapted to employ simple keywords (Supplementary Table 2). Accepted languages were English, German, Russian, Polish, Italian, Dutch, and French. All non-English articles were translated by the authors except for Italian articles which required the assistance of a collaborator (who is mentioned in the Acknowledgments).

### Eligibility Criteria (PICOs)

The following PICOS (Population, Intervention, Control, Outcomes, and Study design) eligibility criteria were applied:

P—Humans, animals, and eukaryotic cell lines.

I—Fasting (> or equal to 12 h) or caloric restriction (reduction of at least 30% of normal intake for > or equal to 12 h) prior to a partial or whole-body exposure to ionizing radiation

C—Same population as in the intervention group, with the same ionizing radiation exposure but without any dietary restriction.

O—Molecular, biochemical (short or long term) or clinical responses (short or long term) to ionizing radiation exposure.

S—Controlled trials

A complete list of all included outcomes is shown in Supplementary Table 3.

### Study Selection

Two reviewers independently assessed the eligibility of the studies based on the PICOs criteria *via* screening performed using the Rayyan web application (34). After duplicates were removed, the initial screening was performed using titles and abstracts. Articles were excluded if the title or abstract did not reveal a direct link to the current eligibility criteria (see PICOs). All remaining articles were then screened as full text. A third, and independent experienced reviewer resolved any disagreements.

### Data Extraction

Data from the included studies were extracted using an adapted version of the Cochrane Collaboration's Data collection form for RCTs and non-RCTs (RCT: randomized controlled trial, version 3, April 2014, https://dplp.cochrane.org/data-extraction-forms). The extracted information included characteristics of the study (authors, design, and publication year), population (age, sex, and species/breed if available), radiation (type, intensity, duration, target, circumstances, and control group), fasting and caloric restriction (duration, intensity, control group, chronology with the irradiation), statistical methods and outcomes (parameters, values, time points).

### Assessment of Study Quality

The quality of included studies was appraised and described by the two reviewers using the Cochrane Collaboration's risk of bias analysis tool (https://www.ncbi.nlm.nih.gov/books/NBK132494/bin/appf-fm1.pdf). Uncertainties or discrepancies were discussed with an independent experienced third reviewer. Risks were scored as “low,” “high,” or “unclear.”

### Data Analysis and Statistics

Effect sizes were calculated for all studies that presented their results as means and standard deviations/standard error of the mean. Presented effect sizes were calculated and bias corrected for potential small sample sizes using the Hedge's g method (35) and they refer for any given effect to be either in favor of the control group, or in favor of the intervention group. For ease of interpretation all outcomes were presented to show a positive effect as being “beneficial,” therefore any original outcomes that have negative “beneficial” effects were inverted by multiplying by −1 for presentation in the overall results (e.g., a fall in resting heart rate results in a negative effect size but it is associated with better general physical fitness and thus is considered a beneficial outcome). Thresholds for effect sizes were defined as 0.1 (small), 0.3 (moderate), 0.5 (large), 0.7 (very large), and 0.9 (extremely large) for comparisons between intervention and control groups (35). Cappelli et al. (36), reported only raw data from which to determine mean time of survival after exposure and its standard deviation. Yoshida et al. (37) and Bonilla et al. (38) reported standard error that was converted to standard deviation *via*: SD = SE x √N. In addition, when results were only presented as figures (38–40), a plot reader was used to extract the data (https://automeris.io/WebPlotDigitizer).

Not all studies provided data that allowed effect size calculations using the Hedge's g method. Although these extracted data are inconsistent with the standard practice of Cochrane systematic reviews, they are reported as they provide valuable information in the context of this review. For data extracted from studies that could not be used in the effect size analysis, the statistical significance (*P* < 0.05) of individual studies was reported. Some of the older studies did not report any significance values and thus were highlighted by “significance not reported.” To meet the criteria for Cochrane systematic reviews this data is not presented in the results section and has been moved to the beginning of the discussion (see section “other noteworthy findings not able to be included in the analysis”). In order to illustrate the potential of the studied interventions, both types of data were merged and analyzed in Excel (Professional plus 2016 edition, Microsoft, US) and the meta data was presented using the SankeyMATIC tool (http://sankeymatic.com/build/).

## Results

### Study Selection

**Figure 1:**
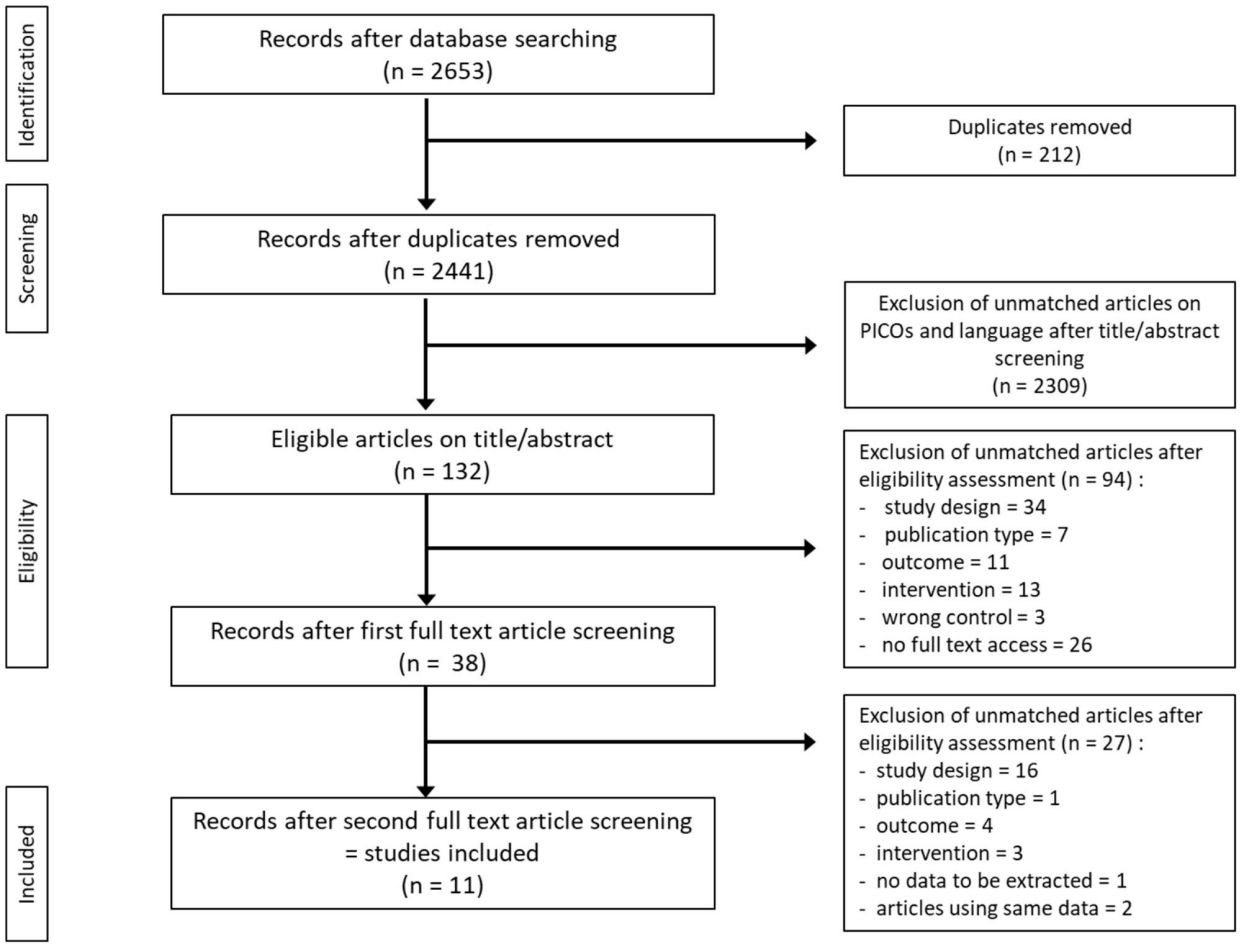
Flow diagram of search and screening methodology. The flow of search results numbers and author's eligibility screening assessment is represented here.

**Figure 2:**
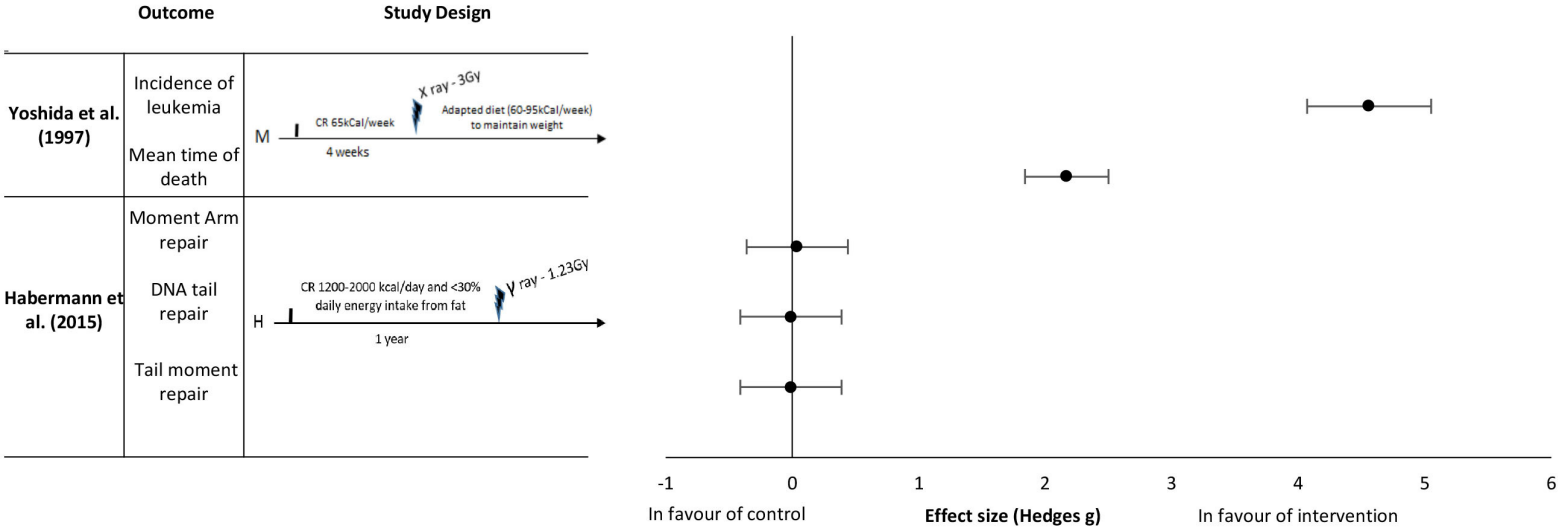
Effect sizes for caloric restriction. Outcomes are plotted with the Hedges' G calculated for each outcome extracted and bias corrected for sample size. Confidence intervals of 95% are represented by the error bars. Effect size values that are in the positive rightward direction indicate a beneficial effect in favor of the intervention group compared to the control group. M, Mice; H, Humans.

**Figure 3:**
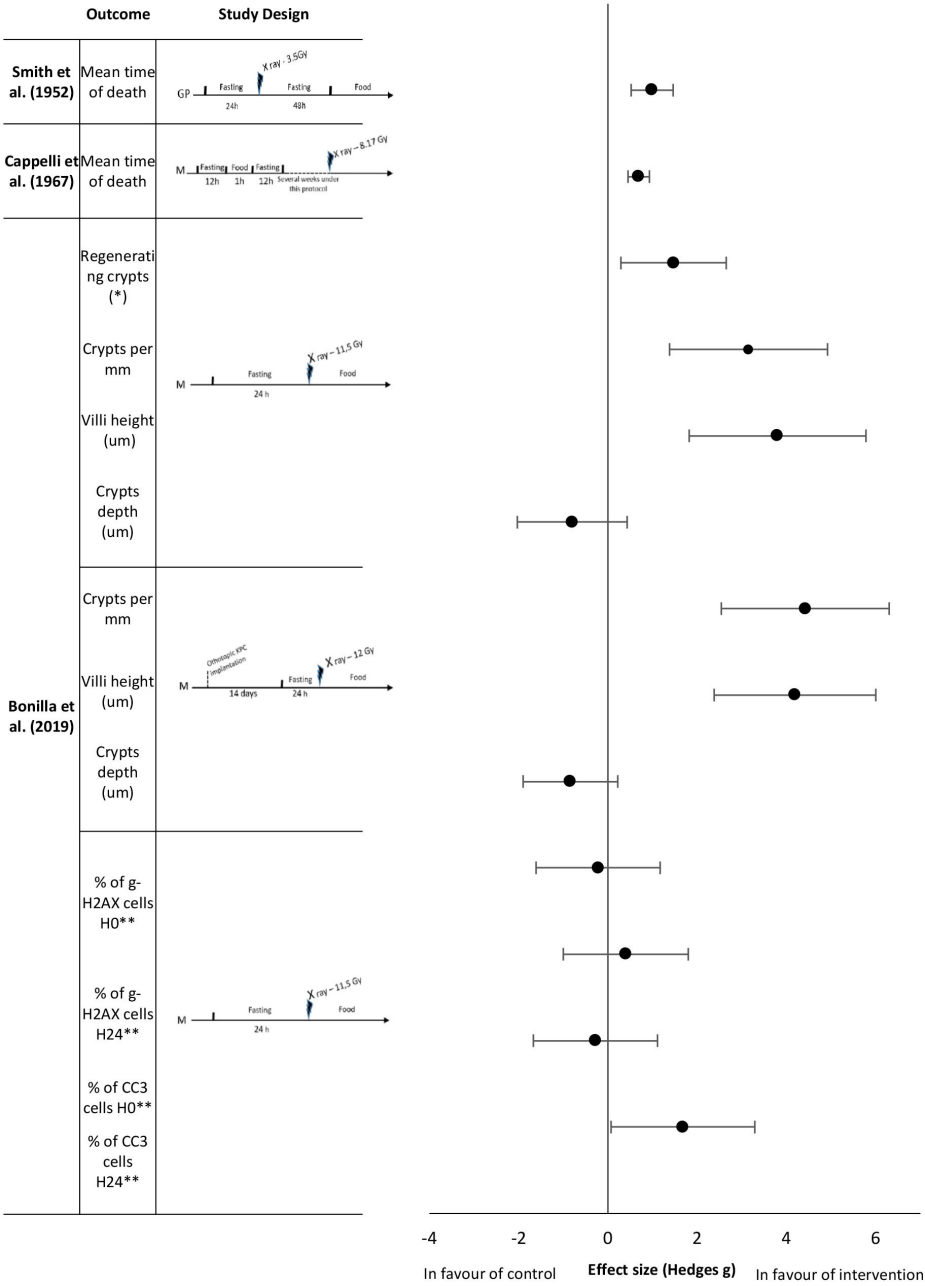
Effect sizes for fasting. Outcomes are plotted with the Hedges' G calculated for each outcome extracted and bias corrected for sample size. Confidence intervals of 95% are represented by the error bars. Effect size values that are in the positive rightward direction indicate a beneficial effect in favor of the intervention group compared to the control group. GP, Guinea pigs; M, mice. *per circumference. ** effect size multiplied by −1.

**Figure 4:**
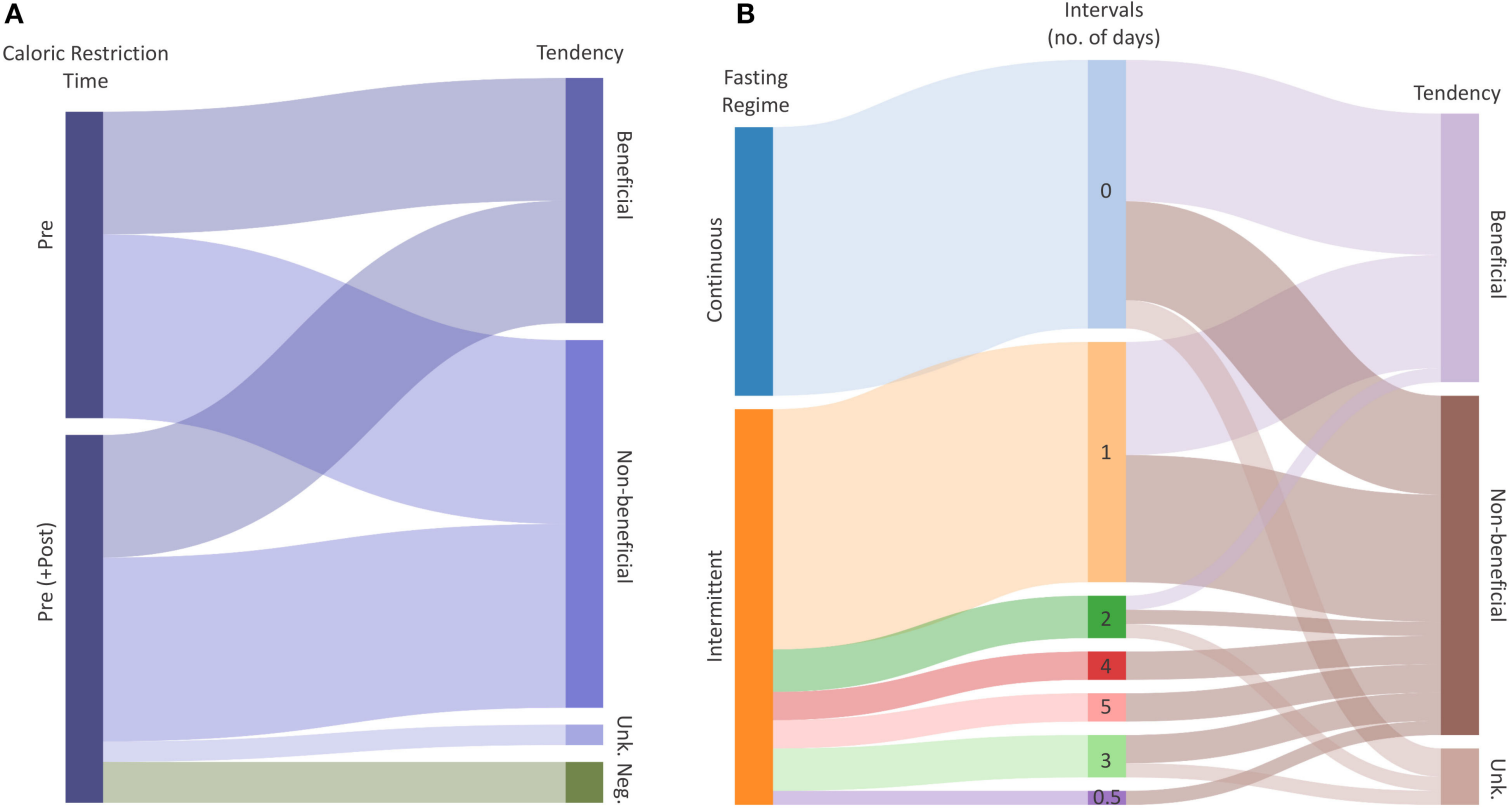
Radio-protective potential of dietary restrictions. **(A)** Caloric Restriction (CR) explained by the time and Tendency of the intervention. Flows represent the total number of outcomes with a certain potential for effectiveness. **(B)** Fasting explained by the regime (Intermittent, Continuous), Intervals and radio-protective effectiveness of the intervention. The middle column represents the duration of one interval in days. Flows represent the total number of outcomes given intervals and tendency, respectively. Tendencies indicate whether the CR or Fasting have radio-protective potential (Beneficial), potentially increase radio-sensitivity (Negative) or have no effect (Non-beneficial). “Unk.” represents an unknown tendency.

The initial search identified 2,441 studies after duplicates were removed. Abstract and title screening excluded 2,309 studies that did not meet the eligibility criteria. This left 132 studies for which attempts to obtain full text versions were made. Since the present selection contained many old studies unavailable electronically, Sorbonne University library network and King's College London Library's support were requested for obtaining physical copies. Additionally, article authors were contacted through ResearchGate (https://www.researchgate.net) and email solicitations to request access to full text articles. After two rounds of full text screening, a further 121 studies were excluded for various reasons, including 26 studies for which full text could not be obtained (listed in Figure 1). Following the screening process, 11 studies, including one RCT and ten controlled trials (CT) were included for analysis (Table 1). Of the 11 included studies five were eligible to calculate effect sizes (Figures 2–4). Studies that did not allow for effect size calculations are presented in the Supplementary Material together with the meta data of all included studies. Included studies mostly reported using animal samples (seven mouse, two rat and one with Guinea Pig). Habermann et al. (41) was the only human study included. All included studies administered either a single high dose of γ-rays (three studies) or X rays (eight studies). There was a high degree of variability on the dosage of radiation delivered to the study population ranging from 1.23 to 12 Gy. Of the 11 studies included, seven enforced fasting and four caloric restriction as an intervention. The outcome parameters varied from clinical (survival rate, leukemia or cancer occurrence) to histological (number of regenerating crypts, number of crypts, villi height in the intestinal mucosa) and biochemical (RNA polymerase activity, DNA repair activity, hematopoietic cell cycle size, and DNA damage markers). A full meta-analysis was not performed due to high heterogeneity in both intervention and reported outcomes across included studies. All the metadata, together with the calculated effect sizes for is available in Supplementary Table 1 (caloric restriction) and Supplementary Table 2 (fasting).

**Table 1:** Overview of all measured outcomes of the 11 included studies.

**Caloric restriction**		**Fasting**	
DNA repair	Habermann et al. (41)	RNA polymerase activity	Omata (40)
Cycle cell in hematopoietic organs	Yoshida et al. (45)	H2AX and CC3 cell expression	Bonilla et al. (38)
		Mucous membrane histology	Bonilla et al. (38)
Tumor growth	Yoshida et al. (46)	Survival rate	Smith et al. (42) Maisin et al. (47) Kozubík and Pospísil (39) Li et al. (48) Bonilla et al. (38)
Leukemia	Yoshida et al. (37) Yoshida et al. (45) Yoshida et al. (46)		
Time of death	Yoshida et al. (37) Yoshida et al. (46)	Time of death	Smith et al. (42) Cappelli et al. (36)

### Methodological Quality

Several studies failed to provide sufficient details to permit a complete assessment of their potential risk of bias (e.g., selection, performance, and bias evaluation). Therefore, these studies were identified as possessing unclear overall risk of bias. All results for the assessment of the methodological quality of the included studies are presented in Supplementary Table 4.

### Effect Sizes of Interventions

#### Caloric Restriction

Yoshida et al. (37) reported an effect in favor of pre-exposure caloric restriction of 65 kCal per week for 4 weeks on the incidence of leukemia with an extremely large effect size of 4.56 and a mean time of death with an extremely large effect size of 2.17 (Figure 2). The mice in this study were kept under a restrictive diet before irradiation, as well as given a moderate caloric restriction after irradiation (reduced caloric intake to maintain weight of 60–95 kCal per week). Habermann et al. (41) studied the repair capacity of radiation damaged DNA *in vitro* following chronic caloric restriction in humans. The intake restriction implemented in this study was 1,200–2,000 kCal/day caloric intake for 1 year before radiation exposure. In this study, no difference was found between the control and intervention groups on the various types of DNA damage (Moment arm repair, DNA tail repair, and tail moment repair) with effect sizes of 0.04, −0.01 and −0.01, respectively (Figure 2).

#### Fasting

To date, literature has focused on short and unique fasting sequences as an intervention prior to irradiation. Smith et al. (42) analyzed the mean time of death of guinea pigs exposed to ionizing radiation after 24 h of fasting, followed by 48 h of fasting after exposure. Fasting improved the mean survival time with an extremely large effect size of 0.99 (Figure 3). Longer fasting sequences were also studied. Cappelli et al. (36) showed that prolonged intermittent fasting with short fasting periods for several weeks increased the mean survival time of mice with a large effect size of 0.69 (Figure 3). Bonilla et al. (38) studied the effects of short term fasting in mice before abdominal irradiation upon a range of histological and biological outcomes. At the intestinal level, all measured histological parameters (number of regenerating crypts, number of crypts, villi height in the intestinal mucosa) showed an extremely large effect size (1.47, 3.15, and 3.80, respectively,) in favor of the fasting group; except for crypts depth analysis which had a large effect size of −0.8 in favor of the control group (Figure 3). The same authors reported no differences in γ-H2AX levels, but they observed that the number of CC3 positive cells in fasted animals was higher 24 h after radiation exposure, with an extreme large effect size of 1.68 (Figure 3). They also studied the same histological parameters (regenerating crypts not studied) with an analogous study protocol that exposed mice to orthotopic pancreatic cancer cell implantation 2 weeks before the fasting period followed by the irradiation. Here, the effect sizes of number of crypts and villi height were extremely large, 4.42 and 4.19, respectively; crypts depth analysis remained showed an effect size of −0.84.

## Discussion

Out of 46 outcomes on fasting and 18 outcomes on caloric restriction, only 13 and 5, respectively, had sufficient data to calculate effect sizes. Furthermore, a high degree of heterogeneity was found in the outcomes, interventions and populations, preventing result pooling and meta-analysis performance. There were large to extremely large effects in favor of pre-exposure fasting in animals for clinical (incidence of leukemia, mean time of death), histological (crypts density, villi height, regenerating crypts) and biological parameters [% of CC3 cells 24 h after irradiation with a 24 h fasting period before irradiation (H24)]. In contrast, effect sizes for crypts depth, % of γ-H2AX at H0 (no fasting)/H24 and % of CC3 cells at H0 were not significant. Caloric restriction results revealed extremely large positive effect sizes for incidence of leukemia and mean time of death in mice, but in association with post exposure weight control. No effect on DNA damage was found in the only human study included in this review between a pre exposure caloric restriction and its control group.

### Evidence Based on Effect Size Calculation

#### Caloric Restriction

Caloric restriction prior to irradiation did not improve the resistance to radiation induced damage in mice or humans, unless it was combined with caloric restriction post-exposure to ionizing radiation. A reduction in the occurrence of leukemia and survival rates in mice if the caloric restriction intervention was given after irradiation (43, 44). According to the authors, a reduction in the oncogenesis pathway rather than the initiation of cancer may be the potential mechanism of cellular adaptation observed with a reduced caloric intake. The evidence outlined above indicates that longitudinal caloric restriction before, during and after irradiation might be needed to observe any significant beneficial results. Thus, the timing and continuity of reduced caloric intake after irradiation seem to be important factors to consider. The studies included in this systematic review employed various reduced caloric intake definitions, both in terms of duration and number of calories provided to the study population. This variability likely had an influence in the outcomes that were assessed. For example, Habermann et al. (41) restricted calories to study participants for 1 year before radiation while Yoshida et al. (37) employed a short-term caloric restriction regime for only 4 weeks before radiation. Despite substantial study design heterogeneity and the data included in this review, the presented evidence suggests that both pre and post ionizing radiation exposure caloric intake restriction may improve median time to death and reduce cancer incidence. However, further studies are needed evaluating duration, timing, and extent of reduced caloric intake in ameliorating biological damage induced by ionizing radiation.

#### Fasting

The main outcomes reported in the studies that employed fasting as an intervention were mean time to death and survival parameters. The studies included in this systematic review suggest that fasting may improve (rat) survival rates and time to death when exposed to ionizing radiation (see Figure 3). These results are consistent with those generally observed with prolonged intermittent fasting in mice although there is variability, depending on mouse strain. In addition responses are likely to be highly dependent upon radiation type e.g., X-Rays and γ-Rays and dosage. However, there is insufficient data, to evaluate these differences. Analogous to the included caloric restriction studies, there was a high degree of experimental (timing, duration, and type of fasting method) heterogeneity. In general the main beneficial outcomes were observed with different types of fasting (continuous, intermittent) as well as timing relative to the radiation (fasting before, fasting before and after), although they may influence outcomes and it has been hypothesized that reduced oxidative stress combined with increased antioxidant activity could explain the observed beneficial outcomes through energy sensing pathways involving Sirtuins, FOXO or TOR (22, 28, 29). However, all fasting studies were in animal models. Thus, further research (including in humans) is required to determine the exact permutation of the duration, type, and timing of fasting that is required to maximize the radioprotective effect, and the underlying mechanisms.

### Other Noteworthy Findings Not Able to Be Included in the Effect Size Analysis

#### Caloric Restriction

Some of the included studies did not report sufficient data to calculate effect sizes. Yoshida et al. (45) investigated a group of mice given only reduced caloric intake pre-irradiation followed by normal food access after irradiation. In this study no difference in the incidence of leukemia was reported. However, when associated with a continued reduced caloric intake after irradiation (weight maintenance), the incidence of leukemia in mice was significantly reduced in comparison to the control group. Thus, in Yoshida et al. (46), only a post-exposure adapted diet of 60–95 kCal/week to maintain weight vs. normal food access after irradiation appeared to have the effect of reduced myeloid leukemia incidence, increased number of tumor free mice and median time of death if the same pre-irradiation caloric restriction protocol of 65 kCal/week for 4 weeks was used (Supplementary Figure 1, Supplementary Table 5). However, no differences were observed for the other tumors.

#### Fasting

Maisin et al. (47) studied the effect of fasting in rats that were irradiated while wearing liver shielding. Fasting significantly improved the survival rate (from 25 to 50%) in liver shielded animals compared to non-fasted animals (Supplementary Figure 2, Supplementary Table 6). No difference was found without liver protection (0% survival in both groups). Omata et al. (40) investigated the activity of Mn^2+^ (Manganese) and Mg^2+^ (Magnesium) dependant RNA polymerase in the liver of mice that were fasted and the non-fasted control group following irradiation. They reported a decrease (significance not reported) in the activity of RNA polymerase in the fasting group compared to the control group when irradiated. A similar result was reported in the non-irradiated groups (fasted and non-fasted), indicating a link between fasting itself and these enzymes (significance not reported). In all these studies, the animals were fasted until 24 h after irradiation with 6.5 Gy of X-ray radiation.

Kozubík and Pospísil (39) investigated the survival rate of several strains of mice exposed to different protocols of intermittent fasting. Fasting improved the survival rate in each group of mice, but the effects varied depending on fasting duration. Survival benefit was seen when the intermittent fasting intervention was longer than 1 week. Additionally, short periods of food access between fasting periods appeared to reinforce the survival effect although no dose effect relationship was highlighted (significance not reported). A more recent study by Li and co-workers (48) investigated short fasting periods in mice (12, 48, and 72 h with eight mice per group) before radiation, which showed a survival rate of 0, 12.5, and 50%, respectively, in comparison to 0% survival in the control group following 7.5 Gy of γ-rays significance not reported. Additionally, Bonilla et al. (38) reported a survival rate of 100% at day 30 in the fasted intervention group and a 0% survival rate in the non-fasted control group (significance not reported).

### Can Caloric Restriction and Fasting Help to Mitigate Radiation Damage?

The present systematic literature review was hampered by the somewhat limited information on the biological effect of ionizing radiation under caloric restrictions and fasting conditions. An attempt was also made to also analyze tendencies in biological responses to ionizing radiation (Figure 4). In doing so, we have tried to identify potential links between the time and duration of the (caloric restriction/fasting-) interventions, and their effectiveness to mitigate radiation damage. We have merged all extracted data including available meta-data (Supplementary Tables 1, 2), and we assigned a “tendency” to each outcome, based on calculated effect sizes or by reported significance levels of the included studies. The collected data of all included studies did not allow to perform a comprehensive meta-analysis, but the analyzed data suggest tendencies toward beneficial effects of caloric restriction and fasting in response to ionizing radiation. For caloric restriction (Supplementary Table 1), the reported tendencies were equally distributed between “beneficial” and “non-beneficial.” Two “negative” tendencies were reported relating to the loss of the circulating and spleen hematopoietic stem cell fractions (45). Such effects may result from differences in control mice at baseline followed by slower rates of proliferation after irradiation. However, these levels did not appear to influence the overall beneficial effect of reduced rates of leukemia in restricted mice. For studies using caloric restriction as an intervention, all reported beneficial tendencies referred to clinical outcomes (Supplementary Table 1).

The included fasting studies reporting biological responses to ionizing radiation revealed in total 47 reported outcomes, with 19 defined as “beneficial,” and 24 being non-beneficial. The included fasting studies employed either intermittent (28 studies) or continuous fasting (19 studies; Figure 3, Supplementary Table 6). Interestingly, our analysis revealed that the majority of outcomes demonstrated a radio-protective effect during the first 3 days of fasting, and then gradually decreasing with each additional fasting day. The data suggests the radio-protective potential of fasting is less dependent on the type, but rather the duration of fasting although the quality of evidence is insufficient to draw conclusions and thus further research is needed to identify the optimal fasting regimes.

#### Quality of Evidence

The overall risk of bias was difficult to estimate due the lack of information. This might have exposed this review to selection bias since the majority of these articles was selected by title only since abstracts were not available. An overview of the Cochrane risk of bias evaluation of all included studies is presented in Supplementary Table 4. Use of a systematic approach minimized the risk of missing studies. However, since it was not possible to use Boolean logic in screening the literature in Space Agency's local databases, searches in these databases had to be limited to single keyword searches, exposing a risk of missing relevant studies. Moreover, the search yielded a significant number of non-English papers that was addressed through translation (with the help of native speakers) of articles in several languages (French, German, Russian, and Italian). Lastly, despite the authors' endeavor to recover full text articles, 26 articles, mostly old, could not be obtained.

### Transferability of Results

Although scientific communities have been paying more attention to studying radiation effects on living organisms, there are serious limitations in transferring findings from *in-vitro*, animal and human occupational and clinical radiotherapy studies to healthy populations, like the population of astronauts (3). Those imperfect analogs differ in radiation qualities, energies, doses and dose rates from the conditions that astronauts will experience during spaceflight. In case of human studies, which mainly comprise of radiotherapy patients, drawing conclusions from populations with greater health risks and subjected to additional treatments may introduce additional bias. Thus, relying on those surrogates restricts the ability to translate radiation knowledge to spaceflight scenarios (3).

This review has several limitations that should be considered when interpreting its findings to humans from the animal (rodent) models used in all but one of the included studies. In animal studies, generally single, whole-body and short radiation exposure is the norm in contrast to short, partial-body and repeated irradiation of fractionated radiotherapy in humans. A plethora of studies has been published on nutrition in patients receiving radiotherapy, however, including radiotherapy patients would include clinical patient populations that would introduce unacceptable levels of confounding factors such as clinical physiological changes, co-morbidities, and pharmacological effects and these confounding factors would substantially decrease or potentially prevent any transferability to the population that we address in the present study. Therefore, most radiotherapy studies did not meet the inclusion criteria for the present study. No data were also available on continuous exposure to whole body ionizing radiation in humans in this systematic review. Nonetheless radiation qualities (x rays and γ-rays) and doses reported in this review are consistent with partial radiotherapy, with cancer occurrence and survival rate being two of the most commonly observed outcomes. However, the present study included a significant number of older studies that lacked complete reporting of methodological details leading to the inability to define risk of bias. Moreover, incomplete or inappropriate (by modern standards) data reporting was a recurrent issue preventing data extraction and effect size analysis.

### Terrestrial Implications

The findings in this review could assist with the goal of preserving healthy tissues while also reducing secondary cancers in clinical radiotherapy. The potentially synergistic positive mechanisms of cellular protection that appear to be provided by fasting and reduced caloric intake may assist with the goal of preserving healthy tissues while also reducing secondary cancers in clinical radiotherapy (49). There is growing evidence that suggests the benefit of fasting in cancer care. Studies have investigated the capacity of fasting to enhance the radiation sensitivity of cancer cells like glioma or breast cancer in mice (50, 51). Thus, a positive effect of fasting on cancer treatment was observed in animals, limiting the damage of surrounding healthy cells and increasing the cancer sensitivity to radiotherapy. If those effects are verified in humans, this could lead to practical considerations to support cancer therapy. Furthermore, similar results with chemotherapy were found in mouse models; short-term fasting has shown to improve chemotherapy efficiency with etoposide (52), mitoxantrone, oxaliplatin (53), doxorubicin, cyclophosphamide and cisplatin (54) in various cancers and protected mice from etoposide-induced lethal DNA Damage (55). Since chemotherapy can cause similar damage to ionizing radiation on cellular level (56), the cross-talk between fasting and stress response needs to be further investigated. Even though no links between fasting and DNA damage were found in the present study, synergies between responses to ionizing radiation and chemotherapy and influence of fasting call for further research. One of the hypotheses to explain the protective effect of fasting would involve better autophagy activity of the immune system (57). Chemo-tolerance seems also to be enhanced in pre-fasted mice with neuroblastoma exposed to etoposide (52). Furthermore, they could potentially be applied in other clinical contexts given the apparent reduction of oxidative stress and upregulation of antioxidant activity that play a part in numerous pathological states (58) such as cardiovascular (59) and neurodegenerative diseases (60).

However, caution should also be taken when applying the findings to potentially vulnerable or frail populations such as oncology patients receiving various modalities of cancer therapies. There are conflicting studies showing detrimental effects of fasting or caloric restriction in frail populations as the risk of cachexia and nutrient deficient states can have negative impacts on wound healing, therapeutic responses, and downstream complications (61–63). Thus, such caloric restrictive or fasting strategies to high risk patients due to possible adverse effects should be applied with caution. Nonetheless, this is an area that requires much more studies before it is implemented to current patients and safety should be the first priority in considering novel adjunctive methods to improve patient outcomes. However, maintaining a long-term caloric restriction can be a difficult task due to issues of compliance. The adherence rates appear greater for intermittent fasting than other traditional nutritional methods like chronic caloric restriction (64). Prolonged intermittent fasting has also been performed over time by various religious cohorts with a relatively high safety profile if exemptions like pregnancy or diabetes are respected (65). Recent studies have also shown that prolonged fasting is safe if done under medical supervision and guidance in selected patients (66). If proven to be effective, diet modification as a concurrent therapy in cancer patients must be implemented with patient safety as a priority in patients who may already be frail. Several studies have already indicated a good profile of tolerance of short-term fasting during chemotherapy in humans (67, 68). Whereas, a short term fasting seems possible to implement, chronic restriction could raise other concerns. Since cancer patients are often exposed to great weight loss, cachexia and sarcopenia due to the cancer itself or as a side effect of treatment (69), a chronic restrictive diet can worsen those parameters and thus clinicians should consider the entire patient's clinical profile, quality of life, and desired outcomes in future trials to assess its efficacy and safety. The current aims of cancer patients' nutrition recommend the optimization of caloric intake quality and quantity (70). Increasing the caloric intake during the allotted eating periods of intermittent fasting schedules to achieve overall recommended daily caloric intake ranges remains a possibility. However, further research is needed to determine the best possible combination of fasting modalities, timing, and duration to achieve maximal beneficial effect while maintaining patient safety. For now, this systematic review points toward a possible link between fasting or reduced caloric intake with improved clinical outcomes. It is hoped that further investigations into the mechanisms and extent of the benefit, such diet modifications, would improve clinical care to achieve better patient outcomes.

### Implications for Human Spaceflight

A number of space agencies have committed to “returning” to the Moon and venturing into deep space. The European Space Agency (representing the ESA member states) together with NASA (the United States), Roscosmos (Russia), CSA (Canada), and JAXA (Japan), have recently announced plans to build the lunar gateway—an orbiting space station around the Moon, that will be a base to support both robots and astronauts exploring the lunar surface (https://www.esa.int/Science_Exploration/Human_and_Robotic_Exploration/Gateway_to_the_Moon). Today astronauts on missions to the International Space Station (ISS) are partially isolated from space radiation environment by the protective shield of the Earth's magnetosphere and atmosphere (71). Leaving these “natural” shields on the way to the Moon would expose astronauts to significantly higher ionizing radiation that differs in regard to its “quality” (composition and energies) as well as its “quantity” (dose) which pose serious threats to human health (3). The risks could be reduced by effective shielding capabilities combined with limited time spent in space, however current shielding materials seem to be insufficient and additionally, they can be sources of ionizing radiation due to spallation events (3, 72). Therefore, there is a need for additional protective radio-protective means such as pharmaceutical and biological countermeasures, as beyond low Earth orbit, astronauts will be exposed to significant doses of primary (solar radiation and galactic cosmic radiation) and secondary ionizing radiation (73). Based on the evidence in this review, prolonged intermittent fasting with short breaks may be a potential biological candidate as a countermeasure mitigating the effects of ionizing radiation. Implementing dietary fasting would be relatively feasible since it is possible to maintain the overall caloric intake of current recommendations by adapting the period of eating (74). Mission planning could also consider the possibility of short term fasting during periods of high solar activity at solar maximums. However, one must also consider the impact of such a strategy on other countermeasures such as physical exercise to prevent musculoskeletal and aerobic astronaut deconditioning (75). However, Winnard et al. (76) recently showed that breaks in exercise of up to 7 days are unlikely to have a major impact on musculoskeletal deconditioning. Thus, with greater understanding of the biological responses to the complex space radiation environment and how to protect against it, dietary restrictions could potentially play an important role in mission planning as a biological countermeasure to radiation damage to ensure the best protection possible for astronauts.

## Conclusion

Whilst the data is incomplete there is some evidence to suggest that fasting and caloric restriction might play a protective role with respect to ionizing radiation in rodents. The radio-protective effect (i.e., lower cancer incidence and greater survival) with caloric restriction was only seen if implemented before and after irradiation whereas the benefits of fasting were seen regardless of timing. The potential application and mechanisms of radioprotection provided by dietary changes could have various applications in both terrestrial and space medicine. However, the transferability of knowledge from animal (rodent) models to humans is questionable and given the paucity of research in the field, these observations are only hypothesis generating. Future research should focus on the role of fasting and reduced caloric intake in humans, in particular examining clinical outcomes in patients undergoing radiotherapy. Furthermore, included studies employed a range of radiation types, doses, and locations which require evaluation and further optimized standardization in future research for the potential benefit in radiotherapy patients and astronauts.

## Data Availability Statement

All datasets generated for this study are included in the article/Supplementary Material.

## Author Contributions

SV and DK: data curation, formal analysis, investigation, methodology, software, and writing—original draft. AF and TW: conceptualization, data curation, formal analysis, funding acquisition, investigation, methodology, project administration, resources, software, supervision, validation, visualization, writing, review, and editing. US and NC: supervision, writing, review, and editing. AW and DG: supervision, writing, formal analysis, data curation, review, and editing. All authors: contributed to the article and approved the submitted version.

## Conflict of Interest

The project was funded by the Space Medicine Team of the European Space Agency (ESA HRE-OM) and KBR GmbH. The funder KBR GmbH provided support in the form of salaries for the authors DG and TW but did not have any role in the study design, data collection, and analysis, decision to publish, or preparation of the manuscript. The remaining authors declare that the research was conducted in the absence of any commercial or financial relationships that could be construed as a potential conflict of interest. The specific roles of all authors are articulated in the author contributions section.
